# Genetic diversity of human respiratory syncytial virus circulating among children in Ibadan, Nigeria

**DOI:** 10.1371/journal.pone.0191494

**Published:** 2018-01-23

**Authors:** Olukunle Ogunsemowo, David O. Olaleye, Georgina N. Odaibo

**Affiliations:** Department of Virology, College of Medicine, University of Ibadan, Ibadan, Nigeria; Louisiana State University System, UNITED STATES

## Abstract

Human respiratory syncytial virus (HRSV) is the most common viral cause of acute lower respiratory tract infections (LRTIs) in infants and young children however, without an effective vaccine licensed for human use till date. Information on the circulating genotypes of HRSV from regions with high-burden of infection is vital in the global efforts towards the development of protective vaccine. We report here the genotypes of HRSV circulating among children in Ibadan, the first of such from Nigeria.Nasopharyngeal and oropharyngeal swabs collected from 231 children presenting with respiratory infections in some health facilities for care as well as those attending immunization centers for routine vaccination in Ibadan, Nigeria were used for the study. The 2^nd^ hypervariable (HVR2) region of the glycoprotein (G) gene of HRSV was amplified and sequenced using HRSV group specific primers. HRSV was detected in 41 out of the 231 samples. Thirty-three of the isolates were successfully subtyped(22 subtype A and 11 subtype B). Fourteen of the subtype A and all the subtype B were successfully sequenced and genotyped. Phylogenetic analysis showed that genotype ON1 with 72 nucleotide (nt) duplication was the major subgroup A virus (11 of 14) detected together with genotype NA2. All the HRSV subtype B detected belong to the BA genotype with characteristic 60nt duplication. The ON1 genotypes vary considerably from the prototype strain due to amino acid substitutions including T292I which has not been reported elsewhere. The NA2 genotypes have mutations on four antigenic sites within the HVR2relative to the prototype A2. In conclusion, three genotypes of HRSV were found circulating in Ibadan, Nigeria. Additional study that will include isolates from other parts of the country will be done to determine the extent of genotype diversity of HRSV circulating in Nigeria.

## Introduction

Human respiratory syncytial virus (HRSV) is the most common viral cause of acute lower respiratory tract infections (LRTIs) in infants, young children, vulnerable adults and immunosuppressed patients. Nearly all children are infected once or multiple times by two years of age [1]. The virus is known to cause repeated infection throughout life, [2–3] perhaps due to immune response that does not last long. Although, the virus was first isolated in 1956 [4], there are currently no safe and effective vaccine licensed for human use.

Human respiratory syncytial virus, previously classified in the genus *Pneumovirus* of the family *Paramyxoviridae* now belongs to the genus *Orthopneumovirus* in the *Pneumoviridae* family [5–6]. It is an enveloped virus with single-stranded, non-segmented, linear RNA genome of negative polarity, approximately 15.2kb in size. The virus has 10 genes that encodes for 11 proteins: NS1, NS2, N, P, M, SH, G, F, M2-1, M2-2, and L [7]. The G and F glycoproteins, which are involved in virus attachment and mediation of viral and cell membrane fusion as well as fusion of infected cell membrane to that of adjacent cell respectively are the major surface antigens that elicit the production of neutralizing antibodies. Human respiratory syncytial virus is divided into two subgroups (A and B), based on antigenic and genetic variations [8]. The major variations between and within the two subgroups are found in the attachment glycoprotein G; a type II glycoprotein, which is heavily glycosylated with N- and O-linked sugars, and contain a cytoplasmic tail, a trans-membrane region and an ectodomain with central conserved region flanked by two hypervariable regions (HVR). Each of the subgroups is further categorized into genotypes based on the phylogenetic analyses of the sequences of the second HVR (HVR2). Subgroup A is categorized into 14 genotypes (GA1 to GA7, SAA1,CB-A, NA1 to NA4 and ON1) [9] while subgroup B is categorized into 27 genotypes (BA1 to BA12, BA-C, SAB1 to SAB4, GB1 to GB4, URU1 to URU2, CB-B, CB-1, BA-CCB and BA-CCA) [10]

Information on the circulating genotypes in high-burdened regions like Nigeria is important for the development of globally protective vaccine. This study was designed to determine the subgroup and genotypes of HRSV circulating among pediatric population in Ibadan, Nigeria.

## Materials and methods

### Sample collection and processing

Samples used for this study were collected in March through to October 2015. The samples were collected from children attending routine immunization clinics in two Primary Health Centers (PHCs) (one each from the five Urban and six semi urban local government) in Ibadan metropolis and showing signs of respiratory infection and those seeking medical care due to respiratory infection at the outpatient clinic or hospitalized at the Our Lady of Apostle Hospital, Oluyoro, a secondary health facility in Ibadan. Signs of respiratory infection was defined as subjective fever or documented temperature of ≥38°C plus any of cough, rhinorrhea, difficulty in breathing and elevated respiratory rate for age [11]. Samples were collected with informed assent from parents / guardian within 7 days of onset of illness from children showing symptoms of upper and / or lower respiratory tract infection. The study protocol was approved by the University of Ibadan/University College Hospital (UI/UCH) ethics committee with approval number UI/EC/14/0284

Both nasopharyngeal and oropharyngeal swabs were collected from 231 children. The swabs from each child were put into the same vial of virus transport medium. The samples were transported on ice to the Department of Virology, College of Medicine, University of Ibadan and RNA extracted. Where RNA extraction was not possible on same day of collection, samples were kept in -80°C until processed. Demographic details of the study participants were collected using structured questionnaire.

### PCR

Viral RNA was extracted directly from the clinical specimen using RNAeasy Mini Viral RNA kit (Qiagen, Hilden, Germany) according to manufacturer’s instruction. cDNA was generated by reverse transcriptase reaction using random hexamer primers and SCRIPT reverse transcriptase (Jena Bioscience, Germany). The template was 5μl of the cDNA in a 25μl PCR reaction mix consisting of DNA Taq polymerase, dNTPs, KCl, and MgCl_2_. Detection of the presence of HRSV in the samples was done using a pair of primer (RSV Forward–GGCAAATATGGAAACATACGTGAA, RSV Reverse—TCTTTTTCTAGGACATTGTAYTGAACAG) that targets the conserved region of the viral matrix gene [12]. Amplification was carried out under the following conditions: 94°C for 2 min, followed by 40 cycles of 94°C for 30s, 53°C for 30s and 72°C for 45s and then a final extension at 72°C for 5min in a conventional PCR procedure. The expected amplified product of 84bp was detected in a 2% agarose gel by electrophoresis. Samples positive for HRSV were further analyzed to determine the virus subtype using HRSV–A and HRSV–B specific primers targeting the second hypervariable region of the G gene as previously described [12] with slight modification.

#### Detection of HRSV A using nested PCR

The first-round PCR was performed with 5 μl cDNA in a 25μl reaction mixture containing 10pmol of each of the primer pairs: RSVA-G513-F AGTGTTCAACTTTGTACCCTGC and RSVA-F131-R CTGCACTGCATGTTGATTGAT. Amplification was carried out at 94°C for 5 min, followed by 40 cycles, each consisting of 94°C for 30s, 58°C for 30s, and 72°C for 1min, then a final extension step of 72°C for 10min. Five microliter of the 1^st^ round PCR product was used as template for second round PCR. The 2^nd^ round PCR was performed in a 25μl reaction mixture with 10pmol of inner primers (Forward: -RSVA-G606-F AACCACCACCAAGCCCACAA and Reverse: -RSV-F22-R CAACTCCATTGTTATTTGCC). The cycling condition was the same as for the 1^st^ round PCR, but for the annealing temperature, which was 53°C. The amplicon with the expected band size of 391bp was detected by electrophoresis in 1.5% agarose gel and visualized using Bio-Rad **Gel Doc™ XR+ System**.

#### PCR for detection of HRSV B

Samples with positive HRSV PCR results were further analyzed for the presence of HRSV-B using PCR. Five microliter of cDNA in a 25μl reaction mixture containing 10pmol of each of HRSV-B specific primer (forward:—BGF–GCAGCCATAATATTCATCATCTCT and reverse:—BGR–TGCCCCAGRTTTAATTTCGTTC) was prepared and incubated in a thermal cycler under the following cycling condition: 94°C for 5 min, followed by 40 cycles of 94°C for 30 s, 63°C for 1 min, and 72°C for 1 min, then a final extension step of 72°C for 10 min. The amplified product with expected band size of 801bp was detected with 1.5% agarose gel electrophoresis and visualized using Bio-Rad **Gel Doc™ XR+ System**.

Samples positive for HRSV, but whose subtype could not be detected with the subtype-specific PCR protocols described above were further analysed by PCR using other sets of primers (G267, F164, ABG490, AG655 and BG517) and conditions previously described by Parveen et al., 2006 and Eshaghi et al., 2012 [13–14]

#### DNA sequencing

The PCR products were purified using a commercially available DNA purification kit (Jena Bioscience PCR Purification kit, Germany) according to the manufacturer’s instructions. The same primers used for the PCR were used to sequence the DNA in the forward and the reverse directions on an ABI Prism 3130 genetic analyzer (Applied Bio Systems). The sequencing reactions were commercially carried out by INQABA BIOTEC, South Africa and Macrogen Europe, the Netherlands. Nucleotide sequences of the second hypervariable region of the G gene obtained in this study were deposited in GenBank and assigned accession numbers KU736767 –KU736784 and MG014703 –MG014709.

### Phylogenetic analysis

Electropherograms were assembled, and consensus sequences were generated using CLC Main Workbench 7.6.2 software (CLC bio, Cambridge, MA, USA). Clustal W 1.6 method in MEGA 5.05 software [15] was used to conduct multiple sequence alignments and phylogenetic analysis of the second hypervariable region of the G gene. Phylogenetic trees were generated using the neighbor-joining method, and the statistical significance of the tree topology tested by bootstrapping with 1,000 replicates on MEGA 5.05. Reference strains of existing HRSV-A and HRSV-B genotypes were retrieved from the GenBank (http://www.ncbi.lm.nih.gov) as at January 16, 2017, and used for tree construction together with the sequences generated in this study. Identical sequences were detected using ElimDupes (http://hcv.lanl.gov/content/sequence/ELIMDUPES/elimdupes.html). Pairwise nucleotide distances were calculated to compare the differences within genotypes of subgroup A and B using MEGA 5.05 and BioEdit sequence alignment editor.

Deduced amino acid (aa) sequences were translated with the standard genetic code using MEGA 5.05. For comparison and identification of amino acid substitution, the second hypervariable region of the G protein of the Nigeria (NGR) sequences of HRSV-A and HRSV-B, generated in this study were aligned with prototype ON1 strain ON6-1210A (GenBank accession number JN257693) and prototype A2 strain (GenBank accession number M11486) for HRSV-A and prototype BA strain BA4128/99B (GenBank accession number AY333364) for HRSV-B.

### Amino acid glycosylation site analysis

Potential N-glycosylation sites were predicted if the encoded amino acids were N-X-T/S, where X was not a proline and accepted if the glycosylation potential was ≥0.5 using NetNGlyc 1.0 server (http://www.cbs.dtu.dk/services/NetNGlyc). The O-glycosylation residues were predicted using the NetOGlyc 3.1 server (http://www.cbs.dtu.dk/services/NetOGlyc-3.1/) and accepted if the G-score was ≥0.5 [10]

## Results

### Detection of HRSV

HRSV was detected in 41 (17.7%) of the 231 samples tested. Out of the 41HRSV-positive samples, 33(80.5%) were successfully subtyped using subtype–specific primers. Twenty-two (53.7%) of the 41 HRSV detected were subtype A, while 11 (26.8%) were subtype B. There was no detection of coinfection with both subtypes.

### Sequence alignments and phylogenetic analysis

Sequences of the second hypervariable region (HVR2) of the G gene obtained from 14 of the subtype A and all of the subtype B were used in phylogenetic analysis. Sequences from 8 of the 22 subtype A samples were unreadable even after several attempts.

**Fig 1:**
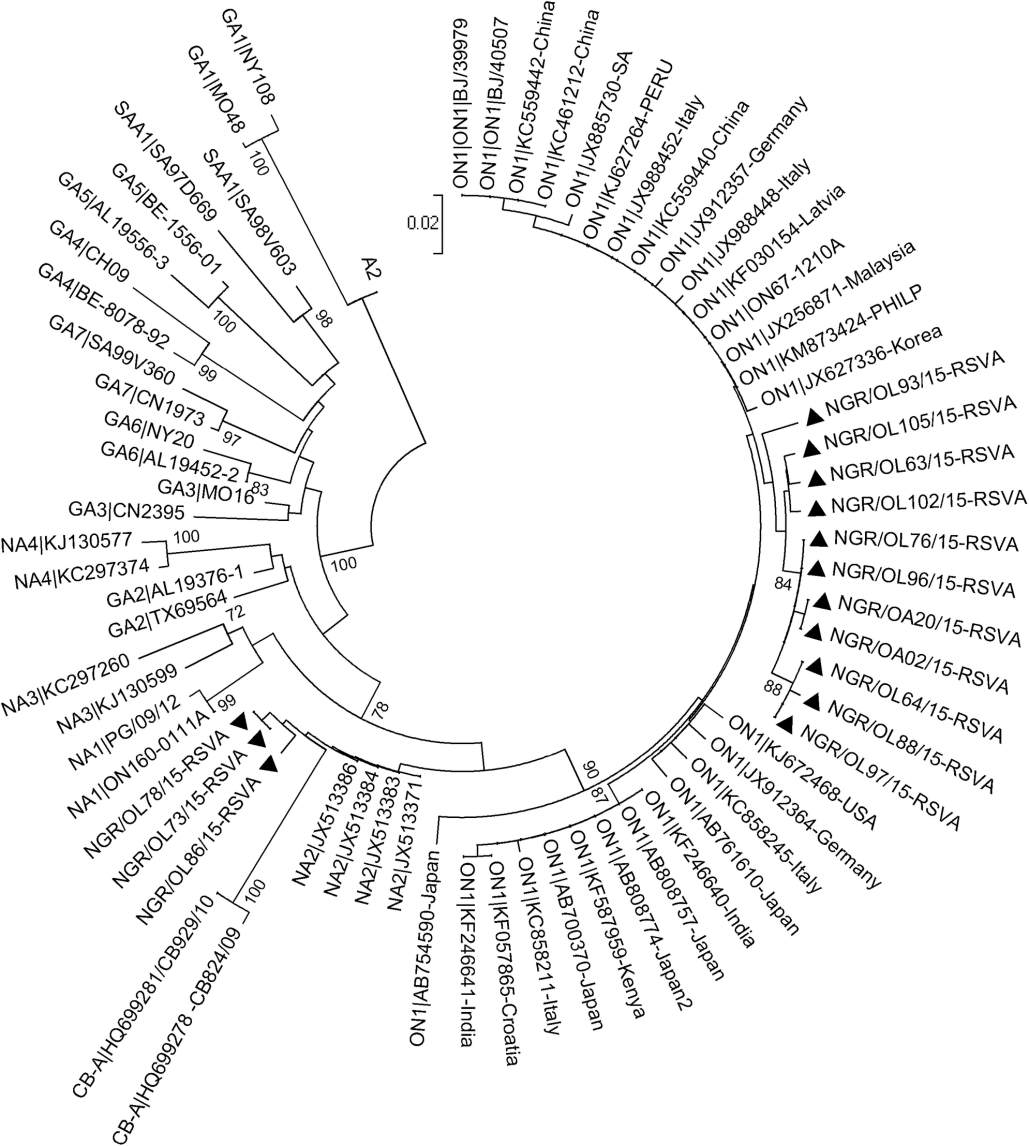
Phylogenetic tree of the second hypervariable region of the G gene of HRSV A. The genotypes represented by the reference strains are indicated before the strain ID with the country of isolation of the ON1 reference strains indicated next to the strain names. The genotypes circulating in Ibadan, Nigeria are indicated by solid triangles. Multiple sequences alignment and phylogenetic tree were constructed using Muscle and neighbor-joining algorithm in MEGA 5.05 software. Statistical significance of the tree topology was tested by 1000 bootstrap replication. Only bootstrap values above 70% are displayed at the nodes. Scale bar indicates nucleotide substitutions per site.

**Fig 2:**
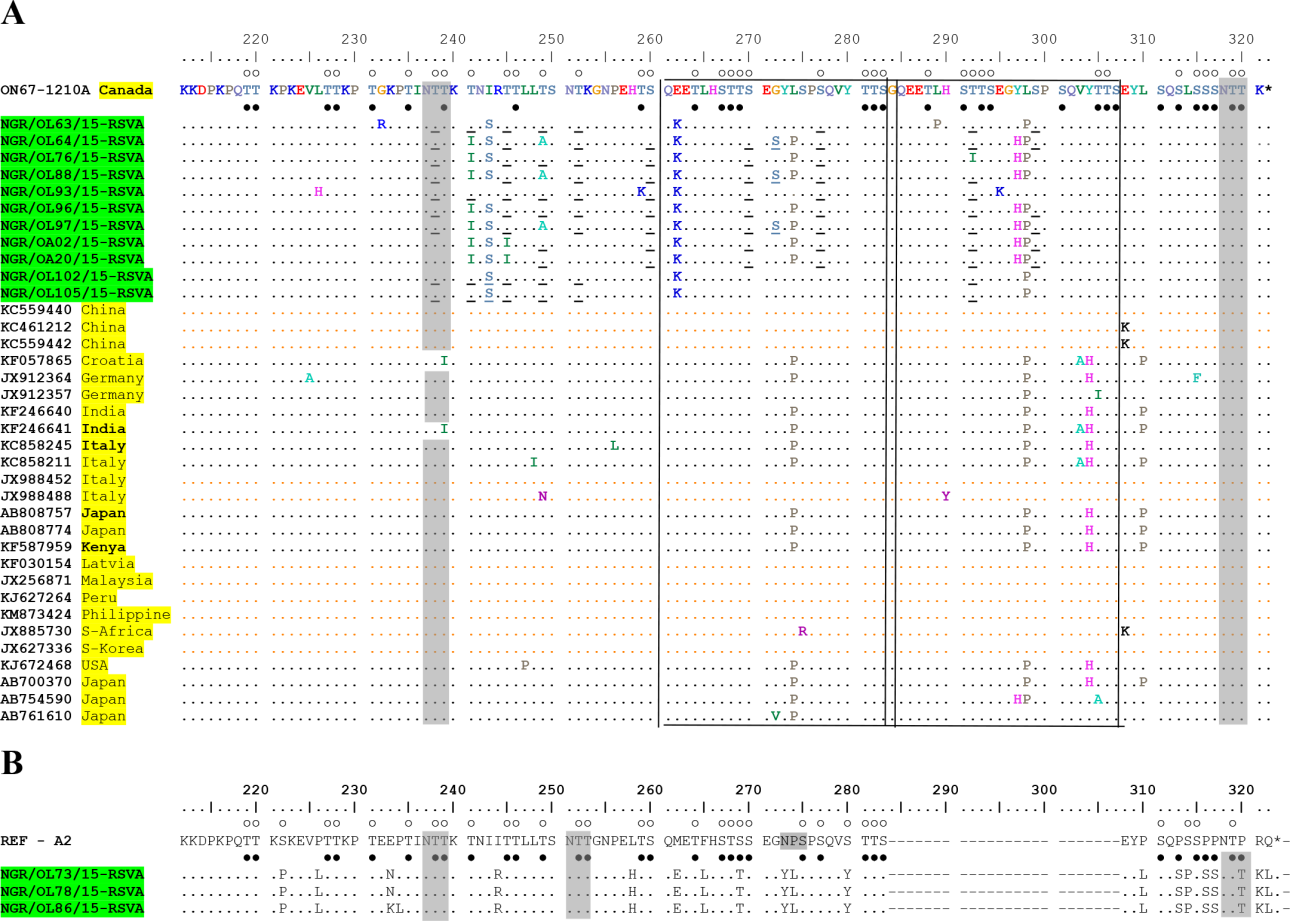
Alignment of deduced amino acid sequences of 2^nd^ hypervariable region of HRSV-A strains. **A)** Alignment of HRSV-A genotype ON1 sequences from different parts of the world. Alignments are shown and residues numbered relative to sequences of prototype ON1 strain ON67-1210A (GenBank accession no. JN257693). The strains from this study are highlighted. The country of isolation of other strains used in the alignment are also highlighted. Identical residues are indicated by dots and stop codons by asterisks. Potential N-glycosylation sites (N-X-T/S, where X is not a proline) are indicated by gray-shaded rectangles. Potential O-glycosylation sites of the prototype ON1 strain are indicated by unfilled circles, while black circles indicated the predicted O-glycosylation sites common in all Nigeria strains. Other predicted O- glycosylation sites, not found in all the strains in Nigeria are underlined. The two copies of the 23 amino acids duplicated sequences are framed by rectangles. **B)** Alignment of HRSV-A subtype NA2 from this study. Alignments are shown relative to the sequence of prototype strain A2 (GenBank accession number M11486). The identifier of strains from this study are highlighted. Residues are numbered relative to the amino acid sequences of prototype ON1 strain ON67-1210A (GenBank accession no. JN257693). Identical residues are indicated by dots, alignment gaps by dashes and stop codons by asterisks. Potential N-glycosylation sites (N-X-T/S, where X is not a proline) are indicated by shaded rectangles. Potential O-glycosylation sites of the prototype A2 strain were indicated by unfilled circles, while black circles indicated the predicted O-glycosylation sites in the Nigeria NA2 strains.

**Fig 3:**
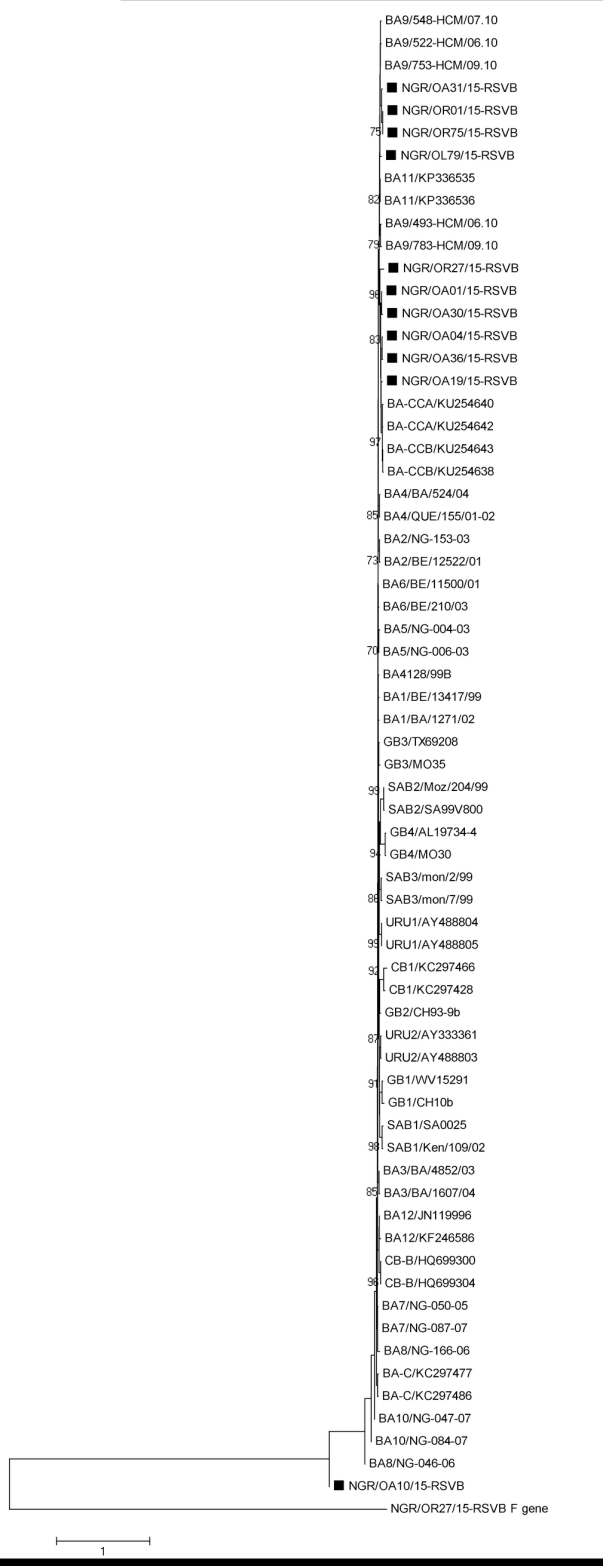
Phylogenetic tree of the second hypervariable region of the G gene of HRSV B. The genotypes represented by the reference strains are indicated before the strain ID. The genotypes circulating in Ibadan, Nigeria are indicated by solid squares. Multiple sequences alignment and phylogenetic tree were constructed using Clustal W and neighbor-joining algorithm in MEGA 5.05 software. Statistical significance of the tree topology was tested by 1000 bootstrap replication. Only bootstrap values above 70% are displayed at the nodes. Scale bar indicates nucleotide substitutions per site.

**Fig 4:**
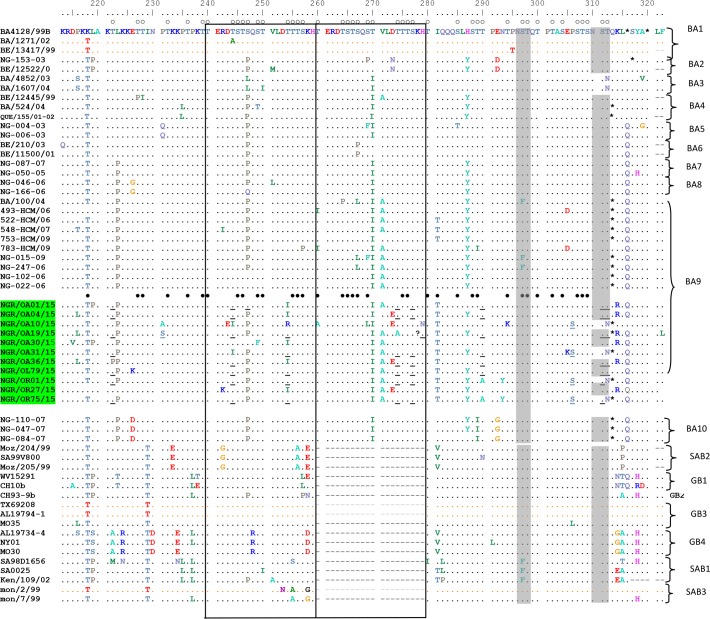
Amino acid alignments of the 2nd hypervariable region of the G protein from HRSV-B. Residues are numbered relative to the sequences of prototype BA4128/99B (GenBank accession number AY333364). Identical residues are indicated by dots; gaps are indicated by dash and stop codons by asterisks. The two copies of the duplicated 20-amino acids are framed by rectangles. Potential N-glycosylation sites (NXT/S, where X is not a proline) are indicated by shaded rectangles. Potential O-glycosylation sites of the prototype BA strain are indicated by unfilled circles, while black circles indicated the predicted O-glycosylation sites common to all Nigeria strains. Other predicted O–glycosylation sites that are not found in all the strains from Nigeria are underlined. The genotypes are shown on the right by brackets.

The phylogenetic analysis showed that 11 out of 14 (78.6%) of the HRSV-A sequences obtained clustered with the ON1 genotype (Fig 1), and possess the distinguishing 72-nucleotide (24aa) duplication in the HVR2 of the G gene (Fig 2), while the other 3 HRSV-A sequences clustered with strains previously assigned to genotype NA2 (Fig 1). All HRSV-B sequences obtained clustered with strains previously assigned to genotype BA (Fig 3) and contained 60-nt (20aa) insertion (Fig 4) in the HVR2. The BA genotypes identified had two different lengths of amino acids: 312aa and 319aa (Fig 4).

The sequence similarity between the HRSV-A ON1 from Nigeria and the prototype ON1 reference strain (accession number JN257693) ranges between 96.5% to 98.8% at the nucleotide level and 92.7% to 97.2% at the amino acid level respectively. The HRSV-A NA2 genotype from Nigeria has 92.3% to 92.5% and 77% to 78.2% similarities with the prototype A2 strain (accession number M11486) at the nucleotide and amino acid level respectively. The overall nucleotide p-distance between the ON1 sequences from Nigeria was 1.8% while that for the NA2 genotype was 0.8%.

The HRSV-B from Nigeria, belonging to the BA genotype had 86.3% to 95.2% and 84.9% to 92.5% similarities with the prototype BA (accession number AY333364) at the nucleotide and amino acid levels respectively. The overall nucleotide p-distance between the BA sequences from Nigeria was 5.6%.

### Deduced amino acid sequence analysis

The alignment of the deduced amino acid sequences of HRSV-A ON1 from Ibadan, Nigeria with sequences previously assigned to ON1 genotype and the alignment of deduced amino acid sequences of HRSV-A, NA2 with prototype A2 strain are shown in Fig 2. The 72nt (24aa) insertions in the ON1 sequences translated into an extension in the amino acid to a length of 321 from the 297 of the reference strain A2 that does not have the insertion. Unlike the prototype ON1 strain (JN257693), the amino acid insertions in the ON1 from this study are not exact duplicates of the preceding 23aa region. Different amino acid substitutions were identified in both copies of the 23aa duplicate compared to the prototype ON1 strain which include; E262K common to all and L298P common to all but one ON1 strains found in this study. G272S was found in three strains, L274P in seven of the 11 ON1 strains, while L289P and T292I were found in single but different strains.

Upon comparison of the NA2 strains with the reference A2 strain, specific amino acid substitutions were identified in the Nigeria strain which include; S222P, P226L, E233N, I244R, L258H, M262E, F265L, S269T, N273Y, P274L, S280Y, P310L, P313S, S314P, P316S, P317S, P320T, R321K and Q322L. The amino acid substitution P310L is within the antigenic site at amino acid position 283–291 relative to HRSV A2, which is equivalent to the aa positions 283 and 308–315 in Fig 2.

The deduced amino acid of the HRSV-B strains from this study, like that of the prototype BA strain has 20aa insertion, resulting in increased length of 312 in some of the strains and 319 in others instead of the 296aa length found in other genotypes of subtype B (Fig 4). The regions of the 20aa duplicates found in this study are not exact repeats as obtained in the prototype BA strain. When compared to the prototype BA strain, substitutions T270I and I281T were found in all the Nigerian BA strains. Other mutations found in the strains from this study include: P216L, K218T, L219P, T222P, L223P, E226K, P231S, T244I, S247P, T254I, V271A, D273E, T274A, H287Y, T290A, N293Y, E305K, P306S and T312N.

### Glycosylation sites

All Nigerian ON1 isolates had two predicted N–glycosylation sites at aa position 237 and 318 (Fig 2A), like the reference ON1 strain. The number and positions of predicted O- glycosylation with G-score >5.0 vary in the ON1 strains. The number varies from 38 to 41 sites. All the amino acid positions with O- glycosylation potentials in the prototype ON1 strain were also predicted O- glycosylation sites in the Nigerian strains. Additional predicted O- glycosylation sites, not found in the prototype ON1 strains were also found in some of the strains (Fig 2A).

The two NA2 strains had three predicted N- glycosylation sites at amino acid positions 237, 251 and 318 (numbered relative to the prototype ON1) (Fig 2B). The amino acid substitution N273Y and P274L led to the loss of N glycosylation site while substitution P320T resulted in acquisitions of an additional N glycosylation site in the Nigerian strains relative to the prototype A2. Thirty-three predicted O- glycosylation sites were found in the NA2 strains. All these sites were also found on the reference A2 strain except four positions i.e aa positions 313, 316, 317 and 320. Three of the positions with predicted O- glycosylation found in the reference A2 strain were not found on the Nigerian NA2 strains. Two of these due to mutation (S222P and S280Y) while the other position had G- score <5.0.

Seven of the eleven BA isolates had two predicted N- glycosylation sites at positions 296 and 310 (Fig 4) like the prototype BA strain. The other four BA isolates with 312aa length had single predicted N- glycosylation sites at position 296 as the amino acid substitution T312N led to the loss of the second N- glycosylation site. The number and positions of predicted O- glycosylation with G-score >5.0 vary in the BA strains, ranging from 42 to 44 sites. Two of the predicted O- glycosylation sites found in the strains which were otherwise not found in the reference strain resulted from mutation K218T and I281T.

## Discussion

In our study, viruses belonging to both subgroups A and B of HRSV were detected and found to be co-circulating in similar population. Phylogenetic analyses showed that genotypes of HRSV-A, including ON1 and NA2 were circulating in Ibadan, with ON1 genotype being the most detected. The ON1 genotype has signature 72nt insertion at the C–terminal end of the attachment glycoprotein and was first reported in Ontario in Canada in the year 2012 [14]. This genotype has been increasingly reported in many countries [9, 16–19] since it was first reported in Canada, and may soon become the predominating HRSV-A globally, going by the reports from different parts of the world[18, 20–22]. As at September 2015, when the last sample identified as ON1 in this study was collected, sequence information available in GenBank showed that this genotype had been reported in about twenty-one countries that spread across almost all the continents of the world [22] and may be circulating undetected or unreported in many other countries.

All the HRSV-B viruses found in this study belong to the BA genotype which was first reported in Buenos Aires, Argentina in 1999 [23] with characteristic 60nt (20aa) duplication in the C-terminal end of the G glycoprotein. Similar to the ON1 genotype, the BA genotype has been detected in many countries with high diversity resulting in sub-genotypes (BA1 –BA12), including recently classified BA-CCA and BA-CCB [10]. Phylogenetic analysis showed that the BA isolates from Nigeria clustered into three groups. Group 1 (NGR/OA31/15-RSVB, NGR/OR01/15-RSVB, NGR/OR75/15-RSVB and NGR/OR79/15-RSVB), Group 2 (NGR/OA01/15-RSVB, NGR/OA04/15-RSVB, NGR/OA19/15-RSVB, NGR/OA30/15-RSVB, NGR/OA36/15-RSVB and NRG/OR27/15-RSVB) and Group 3 (NGR/OA10/15-RSVB). The group one appear to be closely related to the BA genotype isolated from Vietnam and previously assigned to the sub-genotype BA9 [24] albeit with bootstrap value below 70% [25–26]. The group two are closely related to the BA from North-Eastern China previously designated as BA-CCA and BA-CCB[10] though with bootstrap value below 70%. However, nucleotide blast of the sequences of the group 2 in GenBank showed their high relatedness to viruses isolated from China, and assigned to sub-genotype BA9, with accession number KT781370 and KT781379. The third group of the BA isolates from Nigeria did not appear in any cluster with previously assigned BA sub-genotype. However, nucleotide blast result showed its similarity to BA9 isolated from China (KT765088).The BA9 sub-genotype was first identified during the 2006–2007 season in Niigata [27] and was reported to be the predominating HRSV-B globally in the 2009–2010 season [28]. This HRSV-B genotype has also been reported in West Africa, and so far, it is the only HRSV-B genotype reported [19]). The BA genotype from Nigeria vary in their stop codon usage, resulting in variation in the extrapolated length of the G protein to 312aa and 319aa in those using TAA and TAG respectively. This is however not surprising as BA genotypes have been known to be highly variable in their stop codon usage, with previous reports of TAA and TAG [24] as well as CAG [23] stop codon usage.

The deduced amino acid sequences of the 2^nd^ hypervariable region of HRSVA indicated that the ON1 subtype from our study vary considerably from the prototype ON1 due to accumulation of mutations. Although the functional effect of most of the substitutions found is not well known, L274P has been reported to be associated with resistance to neutralizing antibody [29–31]. The substitution G232R found in isolate NGR/OL63/15-RSVA may be similar to G232E suggested to improve the probability of survival of RSV [10]. Similarly, amino acid substitution E262K is known to help RSV evade human immunity, thereby enhancing viral survival [10]s. One ON1 isolate (NGR/OL76/15-RSVA) had the substitution T292I within the 24aa insertion region. As far as can be ascertained, this substitution has not been previously reported, therefore showing the genetic diversity of the strain. Amino acid substitutions also occurred at positions (249, 262, 272, 274) previously predicted to be positively selected [18]. Such substitution are beneficial and are associated with antigenic evolution of HRSV [29,32]

Four different antigenic sites have been previously identified in the HVR2 of HRSV-A and numbered relative to the prototype A2 strain [33]. The NA2 isolates had some point mutations on some of these, which include E233N within the 229–240 region; L258H within the 250–258 region; F289L, S293T and N292Y, all within the region equivalent to 265–273 in the prototype A2 strain; P310L, P313S and S314P within the region equivalent to 283–291 in the prototype strain. These may have important antigenic and immunogenic implication on the viral survival. Similarly, two ON1 isolates (NGR/064/15-RSVA and NGR/OL97/15-RSVA) have amino acid substitution G272S, within the antigenic site 265–273, thereby showing antigenic diversity among the ON1 strains.

The frequency and pattern of O-linked and N-linked carbohydrate side chain is vital in defining the antigenicity of HRSV glycoproteins [34–35]. The strains belonging to the ON1 genotype have relatively similar patterns of N and O—glycosylation to the prototype ON1. On the other hand, substitutions N273Y and P274L led to the loss of an N–glycosylation site which was compensated for by another substitution P320T that resulted in an N–glycosylation gain in the NA2 genotype. While some BA genotypes in Nigeria have two N–glycosylation sites, others have a single glycosylation site as substitution T312N led to the loss of potential N–glycosylation sites in some isolates.

In this study, only a relatively small number of RSV isolates were successfully subtyped and genotyped and samples were collected from only two primary health care centers and one secondary health care facility in Ibadan. Hence, the results may neither be representative of the entire population of Nigeria nor that of Ibadan. Future studies with much larger samples sizes and involving more health care facilities as recruitment sites are needed to accurately draw conclusions about the HRSV epidemiology in the Oyo State and in Nigeria.

Of the 41 samples that were tested positive for RSV, 19.5% (8 samples) could not be subtyped by PCR while 39% (16 samples) could not be genotyped by Sanger sequencing. Although this was likely due to the poor quality of the samples and/or suboptimal storage conditions hampering further analysis, it is therefore not impossible that there were additional HRSV genotypes circulating among the study population which remained undetected in this study because the 2^nd^ hypervariable region of the viral G protein could not be PCR-amplified and sequenced with the primers used in this study. This limitation may be addressed in future studies by next generation sequencing technologies.

In conclusion, genotypes ON1, NA2 and BA of HRSV were found circulating in Ibadan, Nigeria. Additional study that will include isolates from other parts of the country will be done to determine the extent of genotype diversity of HRSV circulating in Nigeria.

## References

[1] Shay DK, Holman RC, Newman RD, Liu LL, Stout JW, Anderson LJ. Bronchiolitis-associated hospitalizations among US children, 1980–1996. JAMA. 1999;282(15):1440–6. 10535434 10.1001/jama.282.15.1440

[2] Scott P, Ochola R, Sande C, Ngama M, Okiro E, Medley G, et al Comparison of strain-specific antibody responses during primary and secondary infections with respiratory syncytial virus. J Med Virol. 2007;79(12):1943–50. 10.1002/jmv.20999 17935184 PMC7612239

[3] Collins PL and Grahams BS. Viral and host factors in human respiratory syncytial virus pathogenesis. J Virol. 2008; 82(5):2040–55 10.1128/JVI.01625-07 17928346 PMC2258918

[4] Blount R. E. Jr, Morris J. A. and Savage R. E. 1956 “Recovery of Cytopathogenic Agent from Chimpanzees with Coryza.” *Proc*. *Soc*. *Exp*. *Bio*. *Med*. 92:544–594. 13359460 10.3181/00379727-92-22538

[5] Amarasinghe GK, Bao Y, Basler CF, Bavari S, Beer M, Bejerman N, et al Taxonomy o the order Mononegavirales. Arch Virol. 2017 1–12. 10.1007/s00705-017-3311-7 28389807 PMC5831667

[6] Human S, Moore ML. How close are we to a respiratory syncytial virus vaccine? Futur Virol. 2016;

[7] Cane P. Molecular epidemiology and evolution of RSV In: Cane P, editor. Respiratory syncytial virus Amsterdam: Elsevier; 2007 p. 89–113.

[8] Mufson M, Orvell C, Rafnar B, Norrby E. Two Distinct Subtypes of Human Respiratory Syncytial Virus. J Gen Virol. 1985;66:2111–24. 10.1099/0022-1317-66-10-2111 2413163

[9] Ren L, Xia Q, Xiao Q, Zhou L, Zang N, Long X, et al The genetic variability of glycoproteins among respiratory syncytial virus subtype A in China between 2009 and 2013. Infect Genet Evol [Internet]. Elsevier B.V.; 2014;27:339–47. Available from: 10.1016/j.meegid.2014.07.030 25109878

[10] Zheng Y, Liu L, Wang S, Li Z, Hou M, Li J, et al Prevailing Genotype Distribution andCharacteristics of Human Respiratory Syncytial Virus in Northeastern China. J Med Virol. 2017;89:222–33. 10.1002/jmv.24640 27448044 PMC5157725

[11] Bigogo GM, Breiman RF, Feikin DR, Audi AO, Aura B, Cosman L et al Epidemiology of respiratory syncytial virus infection in rural and urban Kenya. J Infect Dis. 2013:208 (Suppl 13). S207–S216. 10.1093/infdis/jit489 24265480

[12] Aamir U. B., Muhammad M. A., Hajra S., Syed S. Zahoor Z. and Birjees M. K. 2013 “Molecular Characterization of Circulating Respiratory Syncytial Virus (RSV) Genotypes in Gilgit Baltistan Province of Pakistan during 2011–2012 Winter Season.” *PLoS ONE* 8 9:16–18. 10.1371/journal.pone.0074018 PMC3772930 24058513

[13] Parveen S, Sullender WM, Fowler K, Leftkowitz EJ, Kapoor SK and Broor S. Genetic variability in the G protein gene of group A and B respiratory syncytial viruses from India. J Clin Microbiol. 9 2006;44(9):3055–3064 10.1128/JCM.00187-06 16954227 PMC1594720

[14] Eshaghi A, Duvvuri VR, Lai R, Nadarajah JT, Li A, Patel SN, et al Genetic variability of human respiratory syncytial virus A strains circulating in Ontario: a novel genotype with a 72 nucleotide G gene duplication. PLoS One [Internet]. 2012 1 [cited 2016 Feb 15];7(3):e32807 Available from: http://www.pubmedcentral.nih.gov/articlerender.fcgi?artid=3314658&tool=pmcentrez&rendertype=abstract 10.1371/journal.pone.0032807 22470426 PMC3314658

[15] Tamura K, Peterson D, Peterson N, Stecher G, Nei M, and Kumar S (2011) MEGA5: Molecular Evolutionary Genetics Analysis using Maximum Likelihood, Evolutionary Distance, and Maximum Parsimony Methods. Molecular Biology and Evolution submitted). 10.1093/molbev/msr121 PMC3203626 21546353

[16] Tabatabai J, Prifert C, Pfeil J, Grulich-Henn J, Schnitzler P. Novel Respiratory Syncytial Virus (RSV) Genotype ON1 Predominates in Germany during Winter Season. PLoS One. 2014;9(10):e109191 10.1371/journal.pone.0109191 25290155 PMC4188618

[17] Cui G, Qian Y, Zhu R, Deng J, Zhao L, Sun Y, et al Emerging human respiratory syncytial virus genotype ON1 found in infants with pneumonia in Beijing, China. Emerg Microbes Infect [Internet]. 2013 4 [cited 2016 Mar 10];2(4):e22 Available from: http://www.pubmedcentral.nih.gov/articlerender.fcgi?artid=3639546&tool=pmcentrez&rendertype=abstract 10.1038/emi.2013.19 26038462 PMC3639546

[18] Agoti CN, Otieno JR, Gitahi CW, Cane PA, Nokes DJ. Rapid spread and diversification of respiratory syncytial virus genotype ON1, Kenya. Emerg Infect Dis. 2014;20(6):950–9. 10.3201/eid2006.131438 24856417 PMC4036793

[19] Fall A, Dia N, Cisse EHAK, Kiori DE, Sarr FD, Sy S, et al Epidemiology and molecular characterization of human respiratory syncytial virus in Senegal after four consecutive years of surveillance, 2012–2015. PLoS One. 2016;11(6):1–15. 10.1371/journal.pone.0157163 PMC4912143 27315120

[20] Kim YJ, Kim DW, Lee WJ, Yun MR, Lee HY, Lee HS, et al Rapid replacement of human respiratory syncytial virus A with the ON1 genotype having 72 nucleotide duplication in G gene. Infect Genet Evol [Internet]. Elsevier B.V.; 2014;26(May):103–12. Available from: 10.1016/j.meegid.2014.05.007 24820343 PMC7106136

[21] Panayiotou C, Richter J, Koliou M, Kalogirou N, Georgiou E, Christodoulou C. Epidemiology of respiratory syncytial virus in children in Cyprus during three consecutive winter seasons (2010–2013): age distribution, seasonality and association between prevalent genotypes and disease severity. Epidemiol Infect [Internet]. 2014;142(11):2406–11. Available from: http://www.ncbi.nlm.nih.gov/pubmed/24476750 10.1017/S0950268814000028 24476750 PMC9151279

[22] Duvvuri VR, Granados A, Rosenfeld P, Bahl J, Eshaghi A, Gubbay JB. Genetic diversity and evolutionary insights of respiratory syncytial virus A ON1 genotype: global and local transmission dynamics. Sci Rep [Internet]. Nature Publishing Group; 2015;5(April):14268 Available from: 10.1038/srep14268 26420660 PMC4588507

[23] Trento A, Galiano M, Videla C, Carballal G, García-Barreno B, Melero J a, et al Major changes in the G protein of human respiratory syncytial virus isolates introduced by a duplication of 60 nucleotides. J Gen Virol [Internet]. 2003;84(Pt 11):3115–20. Available from: http://www.ncbi.nlm.nih.gov/pubmed/14573817 10.1099/vir.0.19357-0 14573817

[24] Tran DN, Pham TMH, Ha MT, Tran TTL, Dang TKH, Yoshida L-M, et al Molecular epidemiology and disease severity of human respiratory syncytial virus in Vietnam. PLoS One [Internet]. 2013 1 [cited 2016 Mar 10];8(1):e45436 Available from: http://www.pubmedcentral.nih.gov/articlerender.fcgi?artid=3551923&tool=pmcentrez&rendertype=abstract 10.1371/journal.pone.0045436 23349659 PMC3551923

[25] Venter M, Madhi SA, Tiemessen CT, Schoub BD. Genetic diversity and molecular epidemiology of respiratory syncytial virus over four consecutive seasons in South Africa: Identification of new subgroup A and B genotypes. J Gen Virol. 2001;82(9):2117–24. 11514720 10.1099/0022-1317-82-9-2117

[26] Shobugawa Y, Saito R, Sano Y, Zaraket H, Suzuki Y, Kumaki A, et al Emerging genotypes of human respiratory syncytial virus subgroup A among patients in Japan. J Clin Microbiol. 2009;47(8):2475–82. 10.1128/JCM.00115-09 19553576 PMC2725673

[27] Dapat IC, Shobugawa Y, Sano Y, Saito R, Sasaki A, Suzuki Y, et al New Genotypes within Respiratory Syncytial Virus Group B. J Clin Microbiol. 2010;48(9):3423–7. 10.1128/JCM.00646-10 20610675 PMC2937669

[28] Ohno A, Suzuki A, Lupisan S, Galang H, Sombrero L, Aniceto R, et al Genetic characterization of human respiratory syncytial virus detected in hospitalized children in the Philippines from 2008 to 2012. J Clin Virol [Internet]. Elsevier B.V.; 2013;57(1):59–65. Available from: 10.1016/j.jcv.2013.01.001 23357644

[29] Botosso VF, Zanotto PMDA, Ueda M, Arruda E, Gilio AE, Ieira SE, et al Positive selection results in frequent reversible amino acid replacements in the G protein gene of human respiratory syncytial virus. PLoS Pathog. 2009;5(1). 10.1371/journal.ppat.1000254 PMC2603285 19119418

[30] Martínez I, Dopazo J, Melero JA. Antigenic structure of the human respiratory syncytial virus G glycoprotein and relevance of hypermutation events for the generation of antigenic variants. J Gen Virol. 1997;78(10):2419–29. 9349460 10.1099/0022-1317-78-10-2419

[31] Rueda P, Delgado T, Portela A, Melero JA, Garcia-barreno B. Premature Stop Codons in the G Glycoprotein of Human Respiratory Syncytial Viruses Resistant to Neutralization by Monoclonal Antibodies. 1991;65(6):3374–8. 2033675 10.1128/jvi.65.6.3374-3378.1991 PMC241000

[32] Tapia LI, Shaw CA, Aideyan LO, Jewell AM, Dawson BC, Haq TR, et al Gene sequence variability of the three surface proteins of Human Respiratory Syncytial Virus (HRSV) in Texas. PLoS One. 2014;9(3). 10.1371/journal.pone.0090786 PMC3953119 24625544

[33] Cane PA. Analysis of linear epitopes recognised by the primary human antibody response to a variable region of the attachment (G) protein of respiratory syncytial virus. JMedVirol [Internet]. 1997;51(0146–6615 (Print)):297–304. Available from: file://o/Referenzmanager/Immunglobulin.PDFs/Cane1997.pdf 10.1002/(sici)1096-9071(199704)51:4<297::aid-jmv7>3.0.co;2-0 9093944

[34] Khan WH, Shrungaram RVLN, Broor S, Parveen S. Original Research Article Glycosylation studies of G protein of group B human respiratory syncytial virus (hRSV) in eukaryotic system. 2014;3(10):107–13.

[35] Palomo C, Garcia-Barreno B, Penas C, Melero JA. The G protein of human respiratory syncytial virus: Significance of carbohydrate side-chains and the C-terminal end to its antigenicity. J Gen Virol. 1991;72(3):669–75. 2005433 10.1099/0022-1317-72-3-669

